# The Relationship between the Mechanism of Zinc Oxide Crystallization and Its Antimicrobial Properties for the Surface Modification of Surgical Meshes

**DOI:** 10.3390/ma10040353

**Published:** 2017-03-28

**Authors:** Marta Fiedot, Irena Maliszewska, Olga Rac-Rumijowska, Patrycja Suchorska-Woźniak, Agnieszka Lewińska, Helena Teterycz

**Affiliations:** 1 Faculty of Microsystem Electronics and Photonics, Wroclaw University of Science and Technology, Janiszewskiego 11/17, 50-372 Wroclaw, Poland; olga.rac@pwr.edu.pl (O.R.-R.); patrycja.wozniak@pwr.edu.pl (P.S.-W.); helena.teterycz@pwr.edu.pl (H.T.); 2 Faculty of Chemistry, Wroclaw University of Science and Technology, C.K. Norwida 4/6, 50-373 Wroclaw, Poland; irena.helena.maliszewska@pwr.edu.pl; 3 Faculty of Chemistry, University of Wrocław, Joliot-Curie 14, 50-383 Wrocław, Poland; agnieszka.lewinska@chem.uni.wroc.pl

**Keywords:** zinc oxide, crystallization, antimicrobial properties, free radicals, surgical mesh

## Abstract

Surgical meshes were modified with zinc oxide (ZnO) using a chemical bath deposition method (CBD) at 50 °C, 70 °C, or 90 °C, in order to biologically activate them. Scanning electron microscopy (SEM), mass changes, and X-ray diffraction measurements revealed that at low temperatures Zn(OH)_2_ was formed, and that this was converted into ZnO with a temperature increase. The antimicrobial activity without light stimulation of the ZnO modified Mersilene™ meshes was related to the species of microorganism, the incubation time, and the conditions of the experiment. Generally, cocci (*S. aureus*, *S. epidermidis*) and yeast (*C. albicans*) were more sensitive than Gram-negative rods (*E. coli*). The differences in sensitivity of the studied microorganisms to ZnO were discussed. The most active sample was that obtained at 90 °C. The mechanism of antimicrobial action of ZnO was determined by various techniques, such as zeta potential analysis, electron paramagnetic resonance (EPR) spectroscopy, SEM studies, and measurements of Zn(II) and reactive oxygen species (ROS) concentration. Our results confirmed that the generation of free radicals was crucial, which occurs on the surface of crystalline ZnO.

## 1. Introduction

Hernias, scleral disorders, or vaginal vault diseases are very common and are recurring conditions in children and adults, which calls for an increasing number of surgical procedures. Over the years, various methods have been applied and modified to treat this kind of disease, ranging from the simple surgery of fascia stapling, to laparoscopic techniques and modern materials, such as synthetic meshes [1]. The application of alloplastic implants has decreased the risk of the recurrence of the disease, as well as the intensity of postoperative pain [2]. One of the commonly used implants is Mersilene™, a polyester mesh. It is successfully applied in surgery because it is elastic and its special weave prevents fraying, even in the case of using only selected fragments of this material [3]. Unfortunately, a significant disadvantage of this material is its lack of antimicrobial activity, which leads to the development of postoperative infections. Currently, about 70 percent of the bacteria that cause infections in hospitals are resistant to at least one of the drugs most commonly used in therapy. This problem can be solved by providing the meshes with antimicrobial properties. Such a modification of surgical meshes has not been reported in the literature. In the case of standard synthetic materials, different substances are used e.g., chitosan [4], precious metal nanoparticles [5], and metal oxides micro or nanoparticles [6]. Taking into consideration factors such as simple synthesis, nontoxicity, high biological activity, and low costs, the surface modification of the meshes may be conducted with zinc oxide [7]. It has been widely used in cosmetics and medicaments for many years, because this material in the form of microparticles has been regarded as a safe antibacterial agent.

Zinc oxide can be obtained using different synthesis methods, such as chemical vapor deposition, thermal evaporation, template-directed process, solution-phase synthesis, and hydrothermal precipitation [8]. A significant advantage of this material is that its properties are strongly correlated with its size and morphology. For this reason, zinc oxide particles can be found in the literature in many forms, such as wires, tubes, belts, sheets, and rods [9], on different inorganic [10] or organic [11] substrates. In the case of ZnO application as an antibacterial [12], photocatalytic [13], or sensing material [14], a high surface area is crucial. From this point of view, the most promising application could be one-dimensional particles like zinc oxide rods.

In this article, ZnO was synthesized by a chemical bath deposition process (CBD). In this method, a water solution of zinc ions precursor (most commonly zinc nitrate) and hexamethylenetetramine as a precipitation agent is used. The synthesis is typically carried out in low temperatures, ranging between 50 °C and 95 °C. Although the method has been known for many years, its mechanism is it still not well understood. There are many examples of descriptions of this process indicating different reaction steps, which are based on the equilibrium between the precipitation and dissolution of zinc oxide/hydroxide [15]. It is complicated because every factor of procedure, such as the kind and amount of reagents [16,17], time [18], and the temperature [12] of the reaction, has a significant influence on the obtained particles. It could be concluded that a smaller concentration of zinc nitrate and HMT, resulted in less zinc oxide (hydroxide), with a smaller average number of particles, being deposited on the surface. In this work, only the temperature of the process was changed to indicate the impact of the chemical composition (zinc hydroxide/zinc oxide), morphology, and crystalline structure on the final antimicrobial layer.

Taking into account that the active coating will be deposited on surgical meshes, its action on selected human pathogens will be investigated. In the literature, several descriptions explaining the biological activity of ZnO can be found. In most cases, these are associated with the release of zinc ions [19] or the production of reactive oxygen species (ROS) [20]. It has also been demonstrated that the chemical composition, structure, and morphology have crucial roles in the antimicrobial mechanism [21].

Through the measurements taken in this study, the material characterization of the Zn(OH)_2_/ZnO layer and its biological activity was carried out, in order to perform a careful analysis of the crystallization and antibacterial mechanisms, and to determinate their mutual relationships.

The obtained results are innovative and significantly deepen our knowledge on the generation of antimicrobial properties, not only for synthetic surgical meshes, but also for a range of other materials.

## 2. Results and Discussion

### 2.1. Surface Modification of Mersilene™ Meshes

**Figure 1:**
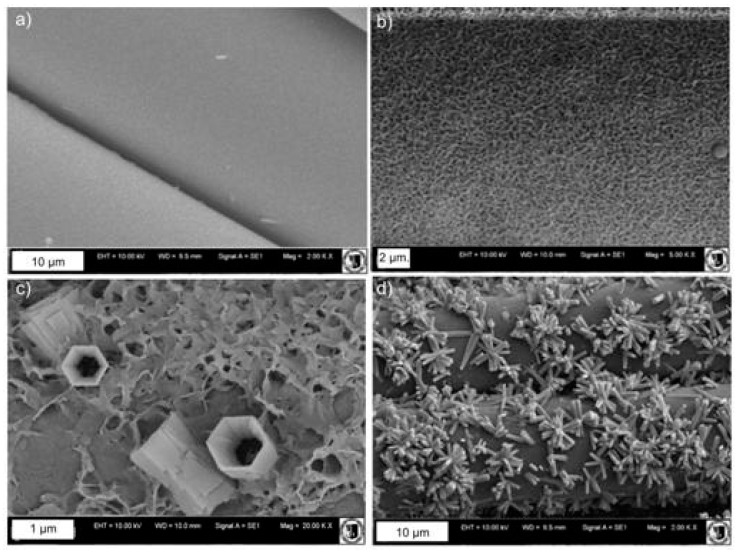
SEM images of meshes: (**a**) pure; (**b**) 50 °C; (**c**) 70 °C; (**d**) 90 °C.

Zinc oxide was deposited on a PET mesh by the CBD method and its antimicrobial activity was then determined. The mesh fiber surface is smooth, as revealed by SEM observations (Figure 1a). Most probably, the amorphous layer of zinc hydroxide is created on the surface of Mersilene™ at 50 °C (Figure 1b). When the temperature increases, the amorphous hydroxide undergoes a change, producing crystalline microstructures (Figure 1c). These microstructures are hexagonal zinc microrods with a diameter of about 450 nm and a height of 1 µm, at 70 °C. Some of the ZnO microrods are hollow. With a further increase in temperature, the amorphous phase completely disappears and hexagonal microrods of zinc oxide are formed (Figure 1d).

The mechanism of the growth of hexagonal ZnO microrods from the solution is complex and involves multiple stages. It is known from the literature that a series of chemical reactions occur during the stirring and heating of zinc nitrate solution and hexamethylenetetramine (Equations (1)–(7)) [22].

(1) $$Zn{\left(NO_{3}\right)}_{2}\to Zn^{2+}+2NO_{3}^{-}$$

(2) $$C_{6}H_{12}N_{4}+6H_{2}O\to 6CH_{2}O+4NH_{3}$$

(3) $$NH_{3}+H_{2}O↔NH_{4}^{+}+OH^{-}$$

(4) $$Zn^{2+}+2OH^{-}↔Zn{\left(OH\right)}_{2}$$

(5) $$Zn{\left(OH\right)}_{2}+OH^{-}↔Zn{\left(OH\right)}_{3}^{-}$$

(6) $$Zn{\left(OH\right)}_{3}^{-}+OH^{-}↔{\left[Zn{\left(OH\right)}_{4}\right]}^{2-}$$

(7) [Zn(OH)4]2−↔ΔTΔTΔTZnO+H2O+2OH−

Richardson et al. showed that ammonia and Zn(II) ions may be found in the solution in different forms, depending on its the temperature and pH. Furthermore, they report that, at a temperature of about 50 °C, a non-ionic form of zinc hydroxide is created; most of which are formed at 60 °C. The compound gradually decomposes and transforms into ZnO at above 80 °C [15].

The growth kinetics of the ZnO structures at different temperatures were observed through an analysis of the weight changes and the XRD measurements of the samples. With the increase in temperature from 50 °C to 70 °C, the weight of the samples gradually increases from 52 to 65 mg ± 1 mg. Above 70 °C, the weight of the deposited layer gradually decreases to 27 mg ± 2 mg. This effect is caused by the increase of the solubility of Zn(OH)_2_ and the rate of ZnO formation. During the transformation of hydroxide to oxide, water is removed from the structure, resulting in a weight decrease of approximately 20% (Equations (5)–(7)). It could be concluded that the growth mechanism of ZnO is correlated with a crystallization and recrystallization process.

**Figure 2:**
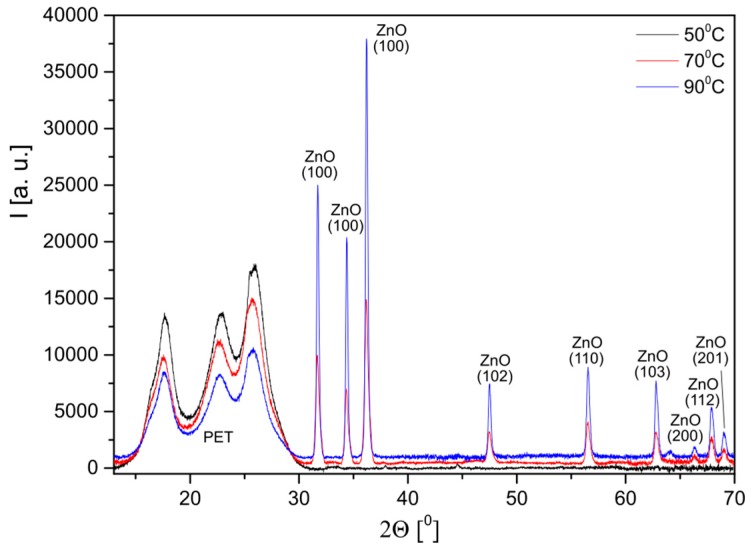
Diffractograms of layers deposited on the polyester meshes at different temperatures.

The crystalline structure of the formed layers was examined by the X-ray diffraction method. No diffraction peaks characteristic of zinc oxide are visible in the diffractograms of the layer formed at 50 °C. They appear in the diffractograms of the layers formed at 70 °C. Their intensity increases with a further increase in the process temperature (Figure 2).

The ZnO has a wurtzite structure of space group C6mc. The interplanar distances for each diffraction line characteristic of ZnO were calculated using Bragg’s law (8), as follows:

(8) nλ=2dsinΘ

where *n*—an integer, *λ*—the length of the X-ray beam, *d*—the lattice spacing, and Θ—the scattering angle. 

Additionally, lattice constants (a, c) and the volume of the unit cell (V) were calculated according to lattice geometry formulas [23]. It was observed that with the increase in temperature, the values of the lattice constants and the volume of the unit cells decreased towards the values given in the literature (Table 1). This means that the crystalline structure of the microrods becomes more orderly. The microstrains and average crystallite size in ZnO were determined by means of the Williamson-Hall (W-H) method (9) [24].

(9) $$\beta_{hkl}\cos\left(\varphi \right)=\frac{k\lambda }{D_{\nu }}+4\varepsilon \sin\left(\varphi \right)$$

where *β*—half-width of the (FWHM) peak, *ϕ*—the angle of the highlight for the given band interference, *k*—the Scherrer constant, *λ*—the length of the X-ray beam, *D_ν_*—the average crystallite size, and *ε*—strain.

**Figure 3:**
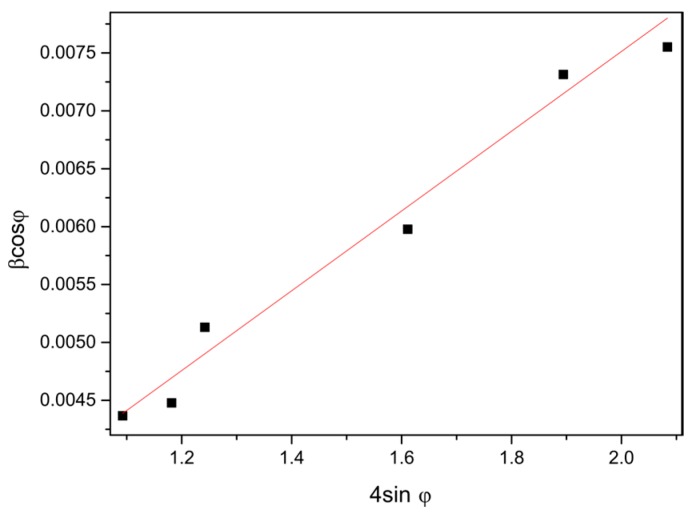
W-H curve for the sample modified at w 90 °C.

This equation is based on the Uniform Deformation Model (UDM). In order to calculate the crystallite size and strain curve, *β*cos*ϕ* = *f*(4sin*ϕ*) was plotted for the preferred diffraction peaks (100), (002), (101), (102), (110), and (103). The W-H curve of zinc oxide produced at 90 °C was demonstrated (Figure 3).

The strain values in the ZnO structure decrease with an increase in temperature. A reverse trend is observed in reference to the crystallites, whose size increases with temperature. The reduction in the strain values and the increase in the size of the crystallites are caused by the arrangement of the crystalline structure (Table 1).

**Figure 4:**
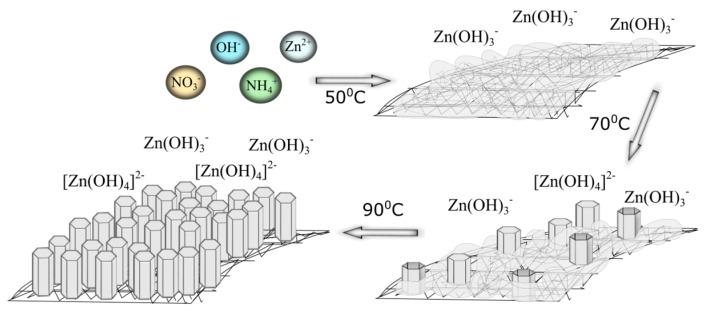
Schema of zinc oxide growth on a mesh surface.

Comparing all of the obtained results with the data in the literature, the authors suggest the following model of the ZnO growth mechanism at different temperatures. In any case, reactions from 1 to 4 occur in the solution. However, the differences occur in the later stages (Equations (5)–(7)). At 50 °C, the amorphous Zn(OH)_2_ partly transforms into Zn(OH)_3_^−^ ions. An increase in the temperature contributes to a partial transformation of the Zn(OH)_3_^−^ into Zn(OH)_4_^2−^, which are the precursors of a further growth of ZnO. At 90 °C, no zinc hydroxide is observed, but the crystalline oxide is directly formed from the solution (Figure 4).

### 2.2. Antimicrobial Properties of the Modified Mersilene^TM^ Meshes

**Figure 5:**
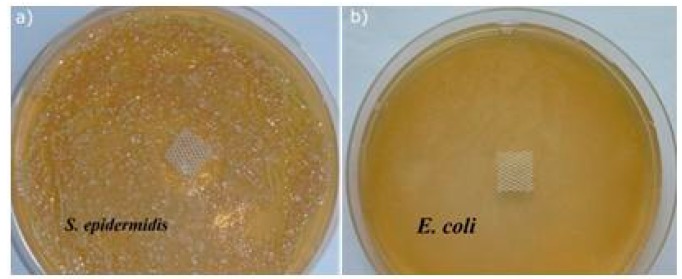
The agar diffusion tests of (**a**) *S. epidermidis* (**b**) *E. coli.*

Studies on the antimicrobial activity of the modified Mersilene™ meshes were carried out against bacteria: *E. coli* (Gram-negative), *S. aureus*, and *S.* *epidermidis* (Gram-positive), and yeast (*C. albicans*). The determination of the antimicrobial properties was conducted on both solid and liquid medium. As shown in Figure 5, the modified Mersilene™ meshes show a negligible antimicrobial activity during a diffusion test. The growth of *S. epidermidis* was only reduced in the case of the mesh prepared at 90 °C (Figure 5). This agar diffusion procedure was only qualitative, and our fundamental experiments were performed by the dilution method. Antimicrobial activity, expressed as the percentage of reduction in viability, was calculated by comparing the size of the initial population with that following the incubation.

Representative results of the reduction in viability for each strain after contact with the ZnO modified Mersilene™ meshes, are presented in Table 2. It can be concluded that *S. epidermidis* was the most sensitive to the ZnO deposited on the Mersilene™ meshes. After 5 h of incubation in physiological saline solution, the mortality rate of these cocci was 44 ± 2%, 85 ± 4%, and 88 ± 3% for the samples produced at 50 °C, 70 °C, and 90 °C, respectively. The ZnO-modified Mersilene™ mesh produced at 90 °C exhibited the best antibacterial activity. After 24 h of contact with this sample, the viable count showed a reduction of 96 ± 3% and 89 ± 3% in the physiological saline solution and liquid broth, respectively.

When *S. aureus* was treated for 5 h with the ZnO-coated Mersilene™ meshes in physiological saline solution, the reduction in the number of living cells was 41 ± 1%, 47 ± 2%, and 56 ± 3% for the samples produced at 50 °C, 70 °C, and 90 °C, respectively. A prolonged contact time of 24 h resulted in the reduction of the viable count to 65 ± 2%, 59 ± 3%, and 72 ± 3%, respectively.

A five-hour treatment of *E. coli* with the ZnO-modified Mersilene™ meshes did not attain a significant killing of cells. Again, a higher mortality rate was observed in physiological saline solution and the reduction in viability was 33 ± 2%, 43 ± 3%, and 47 ± 2% for the samples produced at 50 °C, 70 °C, and 90 °C, respectively. After 24 h of incubation with the meshes, the reduction in viability reached 53 ± 2%, 59 ± 3%, and 63 ± 3%, respectively.

As shown above, the antimicrobial activity of the ZnO deposited on Mersilene™ meshes is related to the species of microorganism, the incubation time, and the experimental conditions. Generally, cocci (*S. aureus*, *S.* *epidermidis*) and yeast (*C. albicans*) are more sensitive than Gram-negative rods (*E. coli*). A drastic reduction in the number of living cells occurred after 24 h of incubation with the ZnO-modified Mersilene™ meshes.

The difference in susceptibility between the Gram-positive (*S. aureus* and *S. epidermidis*) and Gram-negative (*E. coli*) bacteria can be attributed to changes in the interaction mechanisms of ZnO with the bacterial cells. These results overlap with several previous observations. It should be noticed that previous reports mainly refer to ZnO in the form of nanoparticles: Azam et al. showed that ZnO particles were the most effective against Gram-positive bacterial strains (*S. aureus*, *B. subtilis*), when compared to Gram-negative rods (*E. coli*, *P. aeruginosa*) [25]. The authors believed that the differences in the cell wall structure play a significant role. It is well known that Gram-positive bacteria have a much thicker cell wall peptidoglycan compared to Gram-negative bacteria, which results in a decreased susceptibility to the membrane damage induced by ZnO particles. Furthermore, the outer membrane of Gram-negative bacteria can reduce the damage caused by ZnO [26]. Nair et al. [27] suggested that if the antibacterial action mechanism of ZnO particles depends on the generation of reactive oxygen species (ROS), the susceptibility differences are likely to be related to intracellular events, because the membranes of Gram-positive and Gram-negative bacteria are equally permeable to ROS. Another explanation for the increased resistance of *E. coli* to ZnO is the difference in the cell membrane polarization. The membrane of *S. aureus* has a smaller negative charge than *E. coli* [28]. According to Gordon et al. [29], this allows for a greater penetration of negatively charged free radicals such as hydroxyl radicals, and superoxide and peroxide ions, causing damage and cell death in *S. aureus* at concentrations below those required to cause the same effect in *E. coli*.

On the other hand, Applerot et al. [30] found that *E.coli* demonstrated a higher susceptibility to ZnO nanoparticles compared to *S. aureus*. The authors believed that the reason for this may be the difference in the content of the intracellular antioxidant, such as carotenoid pigments, as well as the presence of potent detoxification agents, such as antioxidant enzymes.

As shown in Table 2, *S. aureus* is less sensitive to the ZnO-modified meshes than *S*. *epidermidis*. Several factors may explain this phenomenon. It seems that the cell wall structure is not very significant due to only slight differences in the composition of the cell walls of both staphylococci [31]. The only significant differences are the surface hydrophobicity and charge values. It was previously confirmed, using multiple strains, that *S. aureus* is more hydrophobic than *S. epidermidis*, and that it has a smaller negative charge [32,33,34,35]. Moreover, the carotenoid pigments of *S. aureus* and antioxidant enzymes, particularly catalase, promote more powerful oxidant resistance.

Although metal oxide particles have been widely examined for their antibacterial properties, there are almost no studies regarding their antifungal activity. In the present study, we determined the ability of the ZnO-deposited Mersilene™ meshes to affect the vitality of the pathogenic yeast, *Candida albicans*. It is well known that *C. albicans* is the most common opportunistic fungal pathogen of humans and it is also very common as a hospital-acquired infection [36]. Table 2 shows that, after 24 h of treatment in physiological saline solution, the mortality rate of *C. albicans* was 59 ± 2%, 75 ± 2%, and 85 ± 2% for the samples produced at 50 °C, 70 °C, and 90 °C, respectively. When the liquid broth was used in the experiment, a reduction in the CFU of *C. albicans* was lower and reached 42 ± 3%, 54 ± 4%, and 78 ± 3%, respectively. These results are not in agreement with those obtained by Sawai et al. [37]. These authors found that ZnO powder (at the microscale) exhibits a very weak antifungal activity against *C. albicans*.

A difference in the antimicrobial activity of ZnO-modified Mersilene™ meshes, depending on the experimental conditions, was also observed (Table 2). When our experiments were performed in physiological saline solution (0.85% NaCl), a higher reduction in the viability of the microorganisms was determined. We believe that this difference is due to the presence of proteins in the liquid broth. It was previously demonstrated that MgO particles can kill the bacteria (*E. coli* and *S. aureus*) more efficiently when the medium does not contain proteins [38]. The presence of proteins might have inhibited the adherence of particles to the bacterial cells. Moreover, in the case of the particles coming into contact with components other than bacterial cells, the formed ROS may react with the foreign elements before attacking microbial cells, and thus affect the results of the experiment.

A large effort has been made to explain the antimicrobial action of ZnO, but the exact mechanism of this phenomenon is not clear and is widely discussed. Numerous reports have mainly attributed the bactericidal effect of ZnO particles in the dark to Zn(II) ion dissolution or ZnO internalization [39,40,41,42]. In the case of our study, the internalization of micro Zn(OH)_2_/ZnO particles by living cells is excluded.

The ICP technique was used to estimate the concentrations of zinc ions leached out from micro Zn(OH)_2_/ZnO aqueous suspension during the incubation of the modified Mersilene™ meshes with the living cells. When our experiments were carried out in Nutrient Broth, the amount of Zn(II) ions leached out from the meshes after 24 h was found to be 56.9 ± 0.5 mg/L, 27.6 ± 0.4 mg/L, and 1.0 ± 0.1 mg/L for the samples produced at 50 °C, 70 °C, and 90 °C, respectively. These results show that the concentration of Zn(II) ions released from the meshes made at 50 °C and 70 °C was sufficiently high to inhibit the growth of microorganisms. When studies were conducted in a physiological saline solution, the amount of Zn(II) ions leached out from the Mersilene™ meshes was negligible (from 0.07 ± 0.01 mg/L for the sample made at 90 °C, to 0.3 ± 0.1 mg/L for the sample made at 50 °C).

It is worth noting that there was no significant relationship between the release of Zn(II) ions and the antimicrobial activity of the sample produced at 90 °C. This observation is significant evidence that the leaching of zinc ions can play a major role in the antimicrobial activity of the samples produced at 50 °C and 70 °C (when the experiment was carried out in Nutrient Broth). Yang and Xie showed that proteins (human serum albumin, HSA) stimulate the emergence of zinc ions by the solubilization of ZnO [43]. The authors believed that HSA adsorbs onto the surface of the zinc oxide particles and forms a layer. Then, a complex is formed by the binding of zinc and HSA through C–N groups, and this complex moves into solution, which leads to the dissolution of zinc ions. The vacancies formed during this process become new adsorption sites for HSA. The repetition of this process enhances the dissolution rate of zinc oxide particles. We believe that proteins which are present in Nutrient Broth have a similar function to HSA. It should be noticed that the sample produced at 90 °C displays the highest antimicrobial activity, but the smallest amounts of Zn(II) ions leached from the ZnO particles. This observation automatically leads us to the conclusion that there is another mechanism operating in ZnO which exhibits antimicrobial activity. To better understand this phenomenon, we investigated the possibility of ROS generation.

**Figure 6:**
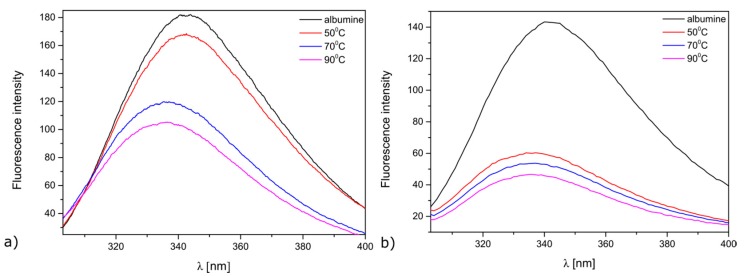
Oxidation of albumin (HSA) in the presence of ZnO-deposited Mersilene™ made at 50°C, 70 °C, and 90 °C after: (**a**) 5 h; (**b**) 24 h.

Firstly, ROS responsible forthe antimicrobial activity of the modified meshes were evaluated through the oxidation of human serum albumin. These studies were carried out in a physiological saline solution. The representative fluorescence spectra are shown in Figure 6. A decrease in the albumin fluorescence emission, peaking at 342 nm, indicates that albumin oxidation occurs. Such a decrease was due to a chemical modification of the aromatic side chains of tryptophyl degradation in the dark, which results in a decrease of tyrosyl residues.

It was observed that that all the samples were able to oxidize HSA (Figure 6). The ZnO-deposited Mersilene™ meshes produced at 90°C showed the greatest efficiency in the oxidation of HAS. The oxidation of albumin was shown to be time dependent and was really significant after 24 h of incubation. Prasanna et al. [44] proposed the mechanism for ROS generation in the dark, involving superoxide species facilitated by surface defects (singly ionized oxygen vacancy) [44]. The authors postulated that atmospheric oxygen can react with an electron from the ZnO surface to form a superoxide radical (^•^O^2−^). A superoxide in water solvates to form a hydroperoxyl radical (^•^HO_2_), and the latter can recombine to form H_2_O_2_. H_2_O_2_ can react with a superoxide radical to form a hydroxyl radical (^•^OH) and a hydroxyl ion (OH^−^). However, singlet oxygen generation is not possible in the dark. 

As was previously mentioned, hydrogen peroxide (H_2_O_2_) and hydroxyl radicals (^•^OH) are mainly responsible for the antimicrobial activity of ZnO. Hydroxyl radicals are negatively charged species that cannot penetrate the cell membrane, whereas hydrogen peroxide can easily penetrate the cell [45]. Therefore, we expect that part of the antimicrobial activity of the ZnO deposited on meshes results from the generation of hydroxyl radicals on the surface of the microbial cell. The production of H_2_O_2_ in a physiological saline solution and in the liquid broth was determined by UV-vis spectrophotometry with KJ and starch [46]. In the present study, a negligible amount of H_2_O_2_ has been revealed (data not shown). Similarly, Prasanna et al. [44] did not detect the generation of H_2_O_2_ by micro ZnO.

**Figure 7:**
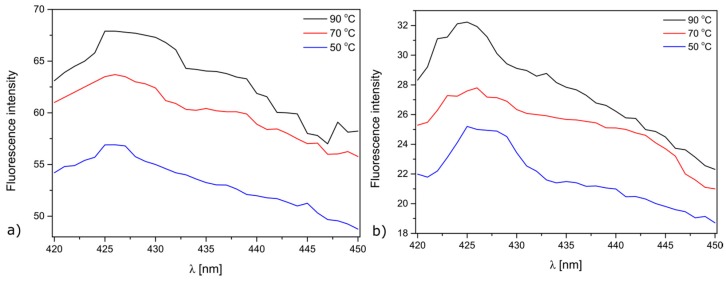
Fluorescence spectra of hydroxyl terephtalic acid in the presence of ZnO-deposited Mersilene™. The experiment was carried out: (**a**) in Nutrient Broth; (**b**) physiological saline solution.

ROS responsible for the antibacterial activity of the modified meshes in the dark was estimated by fluorescence spectroscopy (Figure 7). The fluorescence peak at 425 nm was observed, depicting the spontaneous production of hydroxyl radicals in the dark. These observations are in good agreement with those described by Prasanna [44]. The hydroxyl radical production is much higher in Nutrient Broth than in physiological saline solution.

**Figure 8:**
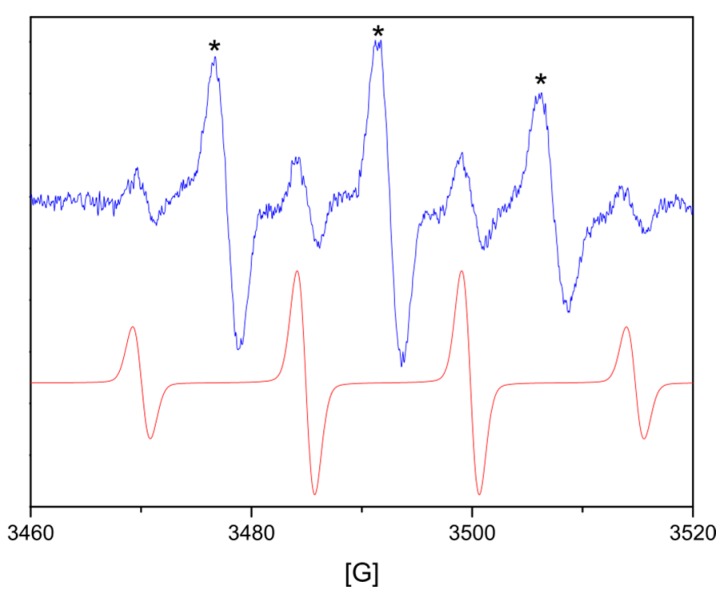
Experimental and simulation EPR spectra of the •DMPO-OH spin adduct of mesh sample made at 90 °C (* nitroxyl radical generated by the oxidation of DMPO).

To further confirm the ROS generated from the samples, EPR spin trapping coupled with DMPO was carried out in the dark. Spin trapping electron paramagnetic resonance (EPR) spectroscopy is one of the most important and reliable techniques for the detection of unstable free radicals. The recorded EPR spectra showed seven lines of various intensity (Figure 8), which clearly suggests the superposition of two spectra due to two different radical species. The spectrum of the first radical exhibits a quartet of lines with a 1:2:2:1 intensity. This spectrum was successfully simulated assuming a_iso_(^14^N) = a_iso_(^1^H) = 14.91 G and g_iso_ = 2.00543. This set of EPR parameters is characteristic of the •DMPO-OH spin adduct, whose formation in the studied system suggests that the HO• radicals are generated from the samples. 

Although the recorded EPR spectra indicated the formation of HO•, it should be noticed that the formation of the O^2−^• radicals on the modified meshes cannot be excluded. DMPO can also trap O^2−^• to produce the spin adduct DMPO-OOH, which is unstable and rapidly decomposes to form a DMPO-OH adduct. The second signal observed on the recorded EPR spectra is composed of three lines with 1:1:1 intensity, and a_iso_(^14^N) = 14.945 G and g_iso_ = 2.00525. It is possible that, due to the high peroxide concentration, DMPO can be oxidized to form a nitroxyl radical other than the DMPO spin adducts [47].

**Figure 9:**
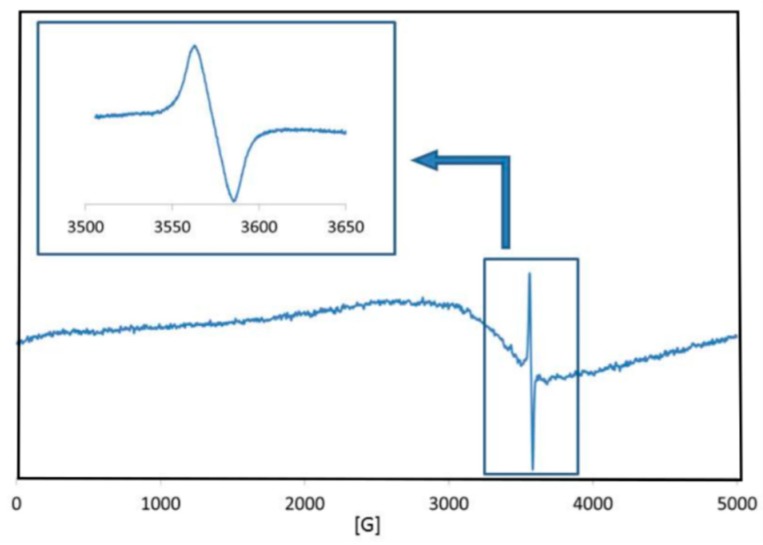
EPR spectra of mesh sample made at 70 °C in the dark.

The experiments were performed in the dark, but atmospheric oxygen can initiate a cascade of reactions. This mechanism of ROS generation in the dark was proposed by Prasanna et al. [44]. The parameters of the DMPO spin adducts of the mesh samples made at different temperatures did not differ much and were in good agreement with those known from the literature [48]. Unexpectedly, in solid diamagnetic-modified Mersilene™, the paramagnetic signal with g-factor ≈ 1.96 was observed. The EPR spectra of the sample produced at 70 °C was demonstrated (Figure 9). A similar result was obtained by Polarz et al., which was interpreted as an attribute of unpaired electrons trapped in oxygen vacancies [49].

In the literature, it has been revealed that a paramagnetic signal often appears, owing to the shallow donor impurities in ZnO, with the g-factor value being practically independent of the type of impurity [50]. In the experiments, it was observed that with an increasing modification temperature of the meshes, the signal of the EPR spectra was clearer.

**Figure 10:**
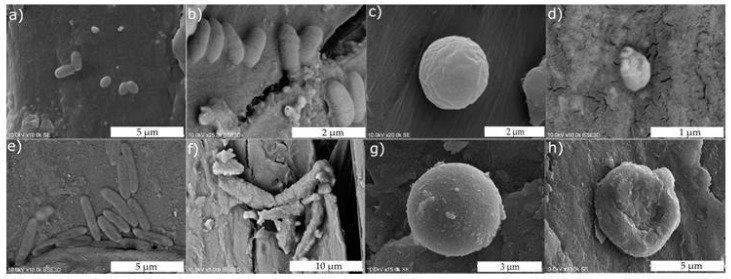
SEM micrographs of: (**a**) untreated *S. aureus*; (**b**) *S. aureus* after ZnO treatment; (**c**) untreated *S. epidermidis*; (**d**) *S. epidermidis* after ZnO treatment; (**e**) untreated *E. coli*; (**f**) *E. coli* after ZnO treatment; (**g**) untreated *C. albicans*; (**h**) and *C. albicans* after ZnO.

The characterization of microbial cell morphology and structure, before and after the contact with ZnO-modified meshes, was determined by an SEM technique (Figure 10a–h). In all cases, it could be seen that, due to the damage of the cell membrane, the intracellular contents leaked out, resulting in cell death. Moreover, a strong elongation of bacterial cells, particularly in the case of *S. aureus* (Figure 10b) and *E. coli* (Figure 10f) after ZnO treating, was observed.

Previously, Zhang et al. [51] and Xie et al. [52] described the interaction between ZnO nanoparticles and bacterial cells (*E. coli* and *C. jejuni*), inducing morphological changes as a result of cell membrane damage. Zhang et al. [53] indicated a strong binding between ZnO particles and the surface of cells, caused by electrostatic forces. The zeta potential of ZnO nanoparticles was around +24 mV at pH 7.0. Therefore, the surface interaction between ZnO and bacterial cells was mainly favoured by the electrostatic forces. In the case of the modified meshes presented in this study, the zeta potential value was −15.7 mV, −32.1 mV, and −22.1 mV for the samples produced at 50 °C, 70 °C, and 90 °C, respectively [54]. When the studies were carried out in Nutrient Broth, the zeta potential value was −11.2 mV, −26.2 mV, and −16.6 mV, respectively. These results provide a reason to believe that a binding between ZnO particles and the bacteria surface due to electrostatic forces is unlikely.

**Figure 11:**
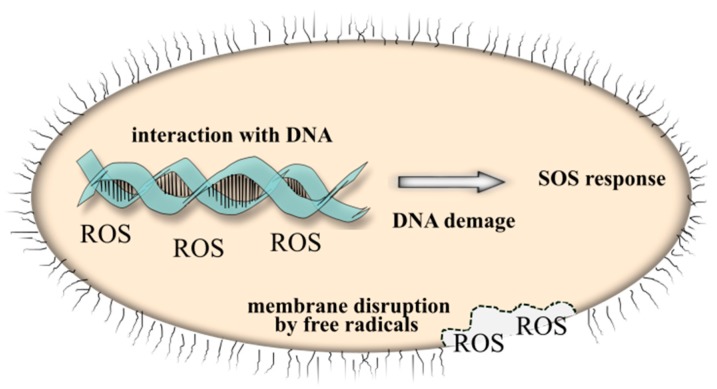
Schema of antimicrobial activity of ZnO.

From all of the studies performed, it could be concluded that the antimicrobial activity of the zinc oxide deposited on meshes is a result of ROS production. The elongation of cells is probably the result of an SOS response, which is a typical bacterial response when a cell’s DNA is damaged after exposure to UV irradiation or oxidative agents [55,56]. Therefore, subjecting bacteria to oxidative stress conditions, such as relatively high concentrations of ROS inside the cell, can activate the RecA protein that causes an induction of the SOS response, and thus, the essential steps in the cell division process are transiently inhibited until the damage to the DNA is repaired (Figure 11).

We thus proved that ZnO on a micro-scale has a significant antimicrobial activity. This discovery is very encouraging, since it is generally considered that ZnO on a micro-scale is safe for use [57]. It is also well known that zinc oxide is widely used in a variety of applications, such as for a UV filter in cosmetic products or a cosmetic colorant. According to the literature, the exposure to nano-scale zinc oxide powders at high doses may cause toxic effects on the hematopoietic system, biochemical system, and liver and kidney [58]. The toxic effects of ZnO nanoparticles are due to their solubility, resulting in an increased intracellular concentration of Zn(II). This results in oxidative stress and the dysfunction of mitochondrion.

## 3. Experimental Section

### 3.1. Structural Characterization

In order to deposit a biologically active layer on the surface of Mersilene™, hydrothermal deposition was used. The process was carried out over a period of 3 h at a temperature of 50, 70, and 90 °C, under an atmospheric pressure in a 100 mM analytically pure equimolar zinc nitrate (V) and hexamethylenetetramine (HMT) solution. The meshes were fixed on glass frames and placed in a beaker with reaction solution. Following this process, the modified materials were rinsed for 5 min with deionized water in the EW-08849-02 Cole-Parmer ultrasound cleaner (Cole-Parmer Instrument Company, Niles, IL, USA), with an effective operating frequency of 55 kHz. Then, the samples were dried in ambient atmosphere. 

The microstructure of the surface-modified Mersilene™ was examined with the use of an electron scanning microscope Hitachi S-3400N, Thermo Scientific Ultra Dry (Hitachi, Hitachi, Japan). The samples were covered with a thin layer of gold to prevent electric charging of the nonconductive samples.

The crystallographic structure of the obtained samples was determined with the use of a Philips Materials Research Diffractometer (Philips, Eindhoven, Netherlands), by means of CuKα radiation. On the basis of the obtained diffractograms, the interplanar distance for each diffraction peak characteristic of ZnO was determined by means of Bragg’s law. In addition, lattice constants (a, c) and the volume of the unit cell were calculated. The average crystallite size and microstrains in ZnO were determined using the Williamson-Hall (W-H) method.

### 3.2. Determination of Antimicrobial Activity

*Staphylococcus aureus* PCM 458, *Staphyloccocus epidermidis* ATCC 49461, *Escherichia coli* PCM 2057, and *Candida albicans* were used as test pathogens to assess the antimicrobial activity of surface-modified Mersilene™.

Their antimicrobial properties were examined using a modified agar diffusion assay (disc test). Microbial inocula were prepared by growing the microorganisms over night in a liquid medium at 37 °C (in the dark). Cultures diluted to approximately 1–1.2 × 10^6^ CFU (colony forming units) per mL were used for preparing a lawn culture using a sterile cotton swab over sterile Nutrient Broth agar. The Petri dishes were kept aside for 5 min. For the qualitative measurement of the antimicrobial activity of surface-modified meshes, the meshes were cut into squares with dimensions of 10 mm × 10 mm, and the antimicrobial properties were examined using a modified agar diffusion assay (disc test) (ISO 20645: 2004, Textile fabrics-determination of antibacterial activity-agar diffusion plate test). The samples were placed on the inoculated Nutrient Broth agar plate and incubated for 24–48 h at 37 °C (in the dark). The inhibition zone was measured as antimicrobial activity against all microbial species. The experiment was repeated thrice, for reproducibility.

The effect of contact time on the reduction of bacteria/yeast was examined by the serial dilution method. For the preparation of inoculum, the overnight culture was centrifuged (5000 rpm; 5 min; 4 °C) and the supernatant was discarded. The cells (pellets) were resuspended in sterilized 0.85% NaCl (physiological saline solution) or in liquid medium (Nutrient Broth). The coated and uncoated (sterilized) meshes (10 mm × 10 mm) were separately placed into the testing tubes and 4 mL of the diluted cells were pipetted into the tubes. One set of experiments was performed with cells suspended in sterilized physiological saline solution; the other was carried out in liquid medium (Nutrient Broth). The initial concentration of microorganisms in the tube was approximately 1.0–1.2 × 10^6^ CFU/mL. The microbial suspension was then incubated at 37 °C (in the dark) and vigorously shaken in order to ensure sufficient contact between the microorganisms and the mesh. After a 5- and 24-h of contact, the number of surviving colonies was determined by plating serial dilution on the plate count agar, to obtain the overall number of microorganisms. The plates were incubated at 37 °C for 24 h and examined for the presence of colonies. This study was carried out separately for each microorganism. In order to evaluate the biocidal activity, the percentage reduction (%) of each test bacterium was calculated using the following formula. The reduction in viability R = (N_0_ − N) × 100/N_0_, where N_0_ and N are the numbers of CFUs at the initial stage (1.0–1.2 × 10^6^ CFU/mL) and those remaining in the suspension for 5 and 24 h in dark conditions, when in contact with Mersilene™ samples. The cultures of the studied microorganisms were incubated under the same reaction conditions which were used as a control. All experiments were run in triplicate and the data acquired is expressed as the average ±SD.

To measure the concentration of Zn(II), the mesh samples (10 mm × 10 mm) were placed in the testing tubes and 4 mL of Nutrient Broth were pipetted into the tube. The second set of experiments was carried out in physiological saline solution. The samples were then incubated at 37 °C (in the dark) and vigorously shaken in order to ensure sufficient contact between the medium and the mesh. After 5 and 24 h of incubation, 1 mL aliquots were drawn out and centrifuged at 8000 rpm, and supernatant liquid was filtered with a membrane (Anapore). The concentration of soluble Zn(II) ions which leached out in the filtrate was estimated by ICP OES, according to the procedure described by Stecka et al. [59]. An inductively coupled plasma sequential spectrometer JY38S (Jobin Yvon, France) was used to measure the concentrations of Zn(II) in all solutions.

The detection of ROS generated from the Mersilene™ samples was performed using the modified method described by Kassab [60] and Bazylińska et al. [61,62]. The ZnO deposited on the mesh (10 mm × 10 mm) was placed in the testing tubes and 4 ml of Nutrient Broth containing albumin (0.8 mg/mL) were pipetted into the tube. The second set of experiments was carried out in physiological saline solution containing albumin (0.8 mg/mL). The incubation was carried out under experimental conditions identical to those described for the dissolution of Zn(II) from the deposited layers. The decrease in albumin concentration as a function of the incubation time was followed by measuring the fluorescence emission in the 300–400 nm range (λ_exc_ = 290 nm), which is typical of the tryptophyl moiety.

H_2_O_2_ generated on the surface of the coated Mersilene™ was estimated by KMnO_4_ redox titrations, according to the method described by Wolanov et al. [63]. The samples (10 mm × 10 mm) were placed in the testing tubes and 4 mL of Nutrient Broth were pipetted into the tube. The second set of experiments were carried out in physiological saline solution. Then, KMnO_4_ and H_2_SO_4_ were added at appropriate concentrations and incubation was carried out under constant stirring at ambient temperature in the dark. After 5 h and 24 h of incubation, the samples were filtered through a membrane filter and H_2_O_2_ was estimated by a standard titration, using a known amount of H_2_O_2_.

Hydroxyl radicals were estimated using fluorescence spectroscopy, according to the procedure described by Xu et al. [46]. The samples (10 mm × 10 mm) were placed in the testing tubes and 4 mL of Nutrient Broth were pipetted into the tube. The second set of experiments was carried out in physiological saline solution. Fluorescence was measured at the excitation wavelength of 312 nm. The intensity of emission at 425 nm is correlated with the hydroxyl radical concentration. Terephthalic acid with hydroxyl radicals forms a 2-hydroxyl terephthalic acid complex, which produces fluorescence with an intensity that is a direct measure of the hydroxyl radical concentration.

To obtain electron paramagnetic resonance spectroscopy measurements, the samples (10 mm × 10 mm) were mixed with spin trap 5,5 dimethyl-1-pyrroline-*N*-oxide (DMPO, 0.02 M), and then transferred into quartz capillaries. The capillaries were placed in the EPR tube and the spectra were recorded on the X-band (~9.7 GHz) EPR-Bruker ELEXYS E500 spectrometer with NMR gaussmeter ER 036TM and E 41 FC frequency counter. A microwave power of 10 mW was used. The presented spectra were accumulated five times at the 1 G modulation amplitude (amplitudes from 0.1–1 G were tested). The samples were measured 10 min after preparation. Computer simulations of the experimental spectra were performed using the WinEPR SimFonia program (1.25 version).

Bacteria and yeast were prepared for SEM analysis by fixation in 2.5% glutaraldehyde. The samples were subsequently treated with phosphate buffer in 2.5% glutaraldehyde, then dehydrated in a series of acetone washes and dried. The SEM measurements were made with Hitachi S-3400N equipped with a tungsten cathode (magnification 80–300.000×) at an operation voltage of 15 keV. Micrographs were acquired with a secondary electron detector (SE) and a backscattered electron detector (BSE).

The zeta potential of the active layers produced at 50 °C, 70 °C, and 90 °C suspended in physiological saline solution and Nutrient Broth (pH 7.2–7.0), was measured by the microelectrophoretic method using a Malvern Zetasizer Nano ZS apparatus. All the measurements were performed at 25 °C. Each value was obtained as an average of three subsequent runs of the instrument, with at least 20 measurements.

## 4. Conclusions

The aim of this study was to design antimicrobial material and to produce a precise examination of the ZnO crystallization process, as well as determine the interdependence between the composition and the structure of the active layer and its antimicrobial activity. 

Only amorphous Zn(OH)_2_ forms on the mesh if the experiments are pursued at 50 °C. If the experiment is completed at 70 °C, ZnO microrods form together with amorphous Zn(OH)_2_, whereas at 90 °C, only ZnO microrods form. That is precisely what was expected based on the literature; we have conformed these predictions by means of SEM and XRD examinations and by determining the estimated active mass. We have also described, in detail, ZnO crystallization on the surface of the polyester mesh.

The measurements of the studied meshes toward *E. coli*, *S. aureus*, *S. epidermidis*, and *C. albicans* demonstrated higher antimicrobial activity during the experiments performed in a physiological saline solution. It was confirmed that cocci and *C. albicans* were more sensitive to ZnO than Gram-negative rods. It was believed that differences in the cell wall structure play a significant role. The highest antimicrobial activity was found when the ZnO-modified Mersilene™ mesh produced at 90 °C was used in the experiments. Taking into account the analyses of antimicrobial activity, the concentration of Zn(II) ions, and ROS generation, it was concluded that zinc ion release, regarded by many authors as one of the antimicrobial mechanisms (in dark), has no significant impact on the antimicrobial properties of the ZnO-deposited Mersilene™ mesh produced at 90 °C. The performed measurements show that the generation of free radicals, which occurs on the surface of crystalline ZnO, is crucial.

The antimicrobial properties of various materials are a subject often described in the literature. In many cases, the cited results are controversial, which calls for procedure methodology which can define these properties. This study deals with the analysis of ZnO biological activity under various experimental conditions (such as liquid broth and physiological saline solution). The obtained results are able to explain the difference in the antimicrobial activities, as described in various reports.

## Figures and Tables

**Table 1:** Changes in ZnO unit cell parameters and crystallites as a function of temperature.

T (°C)	d_(100)_ (Å)	d_(002)_ (Å)	a (Å)	c (Å)	V (Å^3^)	*ε*	D (nm)
50 °C	-	-	-	-	-	-	-
70 °C	2.84	2.63	3.284	5.257	49.116	0.00726	182
90 °C	2.82	2.61	3.259	5.220	48.023	0.00344	220
standard	2.81	2.60	3.250	5.204	47.603	-	-

**Table 2:** Antimicrobial activity of ZnO-deposited Mersilene™ meshes against *E. coli*, *S. aureus*, *S. epidermidis*, and *C. albicans.*

Sample	Duration of Treatment (h)	*E. coli*		*S. aureus*		*S. epidermidis*		*C. albicans*	
		Reduction in Viability (%)							
		s*	NB**	s	NB	s	NB	s	NB
50 °C	5	33 ± 2	22 ± 3	41 ± 1	28 ± 4	44 ± 2	32 ± 2	40 ± 3	37 ± 2
	24	53 ± 2	43 ± 4	65 ± 2	56 ± 3	78 ± 3	67 ± 3	59 ± 2	42 ± 3
70 °C	5	43 ± 3	31 ± 3	47 ± 2	39 ± 2	85 ± 4	70 ± 4	52 ± 4	49 ± 4
	24	59 ± 3	49 ± 2	59 ± 3	46 ± 3	90 ± 3	81 ± 1	75 ± 2	54 ± 4
90 °C	5	47 ± 2	36 ± 3	56 ± 3	53 ± 2	88 ± 3	71 ± 1	80 ± 3	70 ± 4
	24	63 ± 3	57 ± 3	72 ± 3	67 ± 3	96 ± 3	89 ± 3	85 ± 2	78 ± 3

s*: physiological saline solution (0.85% NaCl). NB**: Nutrient Broth.
